# Changes in the left temporal microstate are a sign of cognitive decline in patients with Alzheimer’s disease

**DOI:** 10.1002/brb3.1630

**Published:** 2020-04-27

**Authors:** Christian S. Musaeus, Knut Engedal, Peter Høgh, Vesna Jelic, Arjun R. Khanna, Troels Wesenberg Kjær, Morten Mørup, Mala Naik, Anne‐Rita Oeksengaard, Emiliano Santarnecchi, Jon Snaedal, Lars‐Olof Wahlund, Gunhild Waldemar, Birgitte B. Andersen

**Affiliations:** ^1^ Department of Neurology Danish Dementia Research Centre (DDRC) Rigshospitalet University of Copenhagen Copenhagen Denmark; ^2^ Norwegian National Advisory Unit on Ageing and Health (Ageing and Health) Vestfold Hospital Trust and Oslo University Hospital Ullevaal Oslo Norway; ^3^ Regional Dementia Research Center Department of Neurology Zealand University Hospital Roskilde Denmark; ^4^ Department of Clinical Medicine University of Copenhagen Copenhagen Denmark; ^5^ Division of Clinical Geriatrics Department of Neurobiology, Care Sciences and Society Karolinska Institutet Stockholm Sweden; ^6^ Department of Geriatric Medicine Memory Clinic Karolinska University Hospital Huddinge Sweden; ^7^ Department of Neurosurgery Massachusetts General Hospital Harvard Medical School Boston MA USA; ^8^ Neurophysiology Center Zealand University Hospital Roskilde Denmark; ^9^ Section for Cognitive Systems DTU Compute Technical University of Denmark Lyngby Denmark; ^10^ Department of Geriatric Medicine Haraldsplass Deaconess Hospital Bergen Norway; ^11^ Berenson‐Allen Center for Non‐invasive Brain Stimulation Beth Israel Deaconess Medical Center Harvard Medical School Boston MA USA; ^12^ Department of Geriatric Medicine Landspítali University Hospital Reykjavik Iceland

**Keywords:** Alzheimer's disease, EEG, microstate, mild cognitive impairment, network

## Abstract

**Introduction:**

Large‐scale brain networks are disrupted in the early stages of Alzheimer's disease (AD). Electroencephalography microstate analysis, a promising method for studying brain networks, parses EEG signals into topographies representing discrete, sequential network activations. Prior studies indicate that patients with AD show a pattern of global microstate disorganization. We investigated whether any specific microstate changes could be found in patients with AD and mild cognitive impairment (MCI) compared to healthy controls (HC).

**Materials and methods:**

Standard EEGs were obtained from 135 HC, 117 patients with MCI, and 117 patients with AD from six Nordic memory clinics. We parsed the data into four archetypal microstates.

**Results:**

There was significantly increased duration, occurrence, and coverage of microstate A in patients with AD and MCI compared to HC. When looking at microstates in specific frequency bands, we found that microstate A was affected in delta (1–4 Hz), theta (4–8 Hz), and beta (13–30 Hz), while microstate D was affected only in the delta and theta bands. Microstate features were able to separate HC from AD with an accuracy of 69.8% and HC from MCI with an accuracy of 58.7%.

**Conclusions:**

Further studies are needed to evaluate whether microstates represent a valuable disease classifier. Overall, patients with AD and MCI, as compared to HC, show specific microstate alterations, which are limited to specific frequency bands. These alterations suggest disruption of large‐scale cortical networks in AD and MCI, which may be limited to specific frequency bands.

## Graphical abstract

Large‐scale brain networks are disrupted in the early stages of Alzheimer's disease. Due to the timescale, electroencephalography has shown to be a suitable technique to investigate these cortical networks. By using EEG, we found that large‐scale cortical networks associated with the temporal lobes were disrupted in patients with Alzheimer's disease.

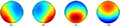

## 1 INTRODUCTION

Large‐scale functional brain networks are altered in patients with Alzheimer's disease (AD) (Dickerson & Sperling, 2009), even in a very early stage of the disease (Cummings, 2004; Selkoe, 2002). Such alterations are considered crucial elements of the neuropathological cascade characterizing AD (Palop & Mucke, 2016). Multiple methods to investigate such brain networks have been proposed, the most common being functional magnetic resonance imaging (fMRI) (Buckner et al., 2005; Greicius, Srivastava, Srivastava, Reiss, & Menon, 2004). However, cortical network dynamics occur at a timescale order of magnitude faster than the blood oxygen level‐dependent signal used in fMRI. The fine temporal resolution of electroencephalography (EEG) indicates that this method may be better suited for studying the fine temporal dynamics of distributed cortical network activity.

One technique for studying distributed brain networks using EEG is microstate analysis, which involves dividing the EEG signal into a number of distinct states (Lehmann, Ozaki, Ozaki, & Pal, 1987) defined by spatial topographies of electric potentials recorded at scalp level. Such parcellation in functional states has been shown to be reliable over multiple recordings (Khanna, Pascual‐Leone, Pascual‐Leone, & Farzan, 2014) and correlated with cognitive abilities (Santarnecchi et al., 2017). It is suggested that spontaneous EEG microstates reflect the activity of resting‐state networks (Van de Ville, Britz, & Michel, 2010; Yuan, Zotev, Zotev, Phillips, Drevets, & Bodurka, 2012), and alterations in the structure and temporal representation of microstates have been documented in other diseases, such as frontotemporal dementia (Nishida et al., 2013) and schizophrenia (Andreou et al., 2014; Lehmann et al., 2005), underpinning it as a potential novel classifier of disease in neurological and psychiatric disorders.

The studies investigating the alterations in microstates in patients with AD (Dierks et al., 1997; Ihl, Dierks, Dierks, Froelich, Martin, & Maurer, 1993; Musaeus, Nielsen, Nielsen, & Hogh, 2019; Nishida et al., 2013; Stevens & Kircher, 1998; Strik et al., 1997) have generated varied and, to some extent, conflicting results. Most studies of AD and healthy controls (HC) find a shorter average duration of microstates in patients with AD (Dierks et al., 1997; Stevens & Kircher, 1998; Strik et al., 1997), although some studies report a longer duration (Ihl et al., 1993; Musaeus, Nielsen, et al., 2019) and one study did not find any significant differences (Nishida et al., 2013). Although examined inconsistently, the microstate syntax may be temporally disorganized in patients with AD (Koenig, Studer, Studer, Hubl, Melie, & Strik, 2005; Nishida et al., 2013). The various and conflicting findings in previous studies are likely due to methodological differences (Dierks et al., 1997; Ihl et al., 1993; Stevens & Kircher, 1998; Strik et al., 1997) and a low number of study participants. Furthermore, in patients with AD, the changes in spectral power have previously been shown to be most prominent in the lower frequencies (Musaeus, Engedal, et al., 2018). However, studies have to our knowledge not investigated the frequency‐specific microstates in patients with AD.

In the current study, we employed a standardized, validated approach to EEG microstate analysis in a large prospectively recruited cohort to investigate changes in microstates in patients with AD and mild cognitive impairment (MCI) compared to healthy aging. Furthermore, we investigated the frequency‐specific microstate changes in patients with AD. In addition, we sought to determine whether microstates could be used as a diagnostic tool for AD and MCI using the same type of classification method as previously described (Musaeus, Engedal, et al., 2018; Musaeus, Engedal, Hogh, et al., 2019). Lastly, we wanted to examine whether microstates correlated with clinical scores or cerebrospinal fluid biomarkers.

## 2 MATERIALS AND METHODS

### 2.1 Participants

The cohort used in the current study was recruited as part of a validation study (Engedal et al., 2015). Spectral power and connectivity analyses are presented elsewhere (Musaeus, Engedal, et al., 2018; Musaeus, Engedal, Hogh, et al., 2019). Participants were recruited from six Nordic memory clinics, and each clinic had to include a minimum of 60 patients and 20 HC who were elderly. In most clinics, all the included patients (*n* = 365) were recruited during their first assessment using the following three predefined exclusion criteria: (a) neurological disorders with dementia other than AD, Parkinson's disease dementia, and Lewy body dementia; (b) major psychiatric disorders; and (c) alcohol or drug abuse. The HC (*n* = 146) were recruited from among family members of the patients, through advertising, or were employees at the recruiting hospitals. All participants gave written informed consent to participate in the study. The study was approved by the ethic committees from Norway, Sweden, Iceland, and Denmark.

**FIGURE 1:**
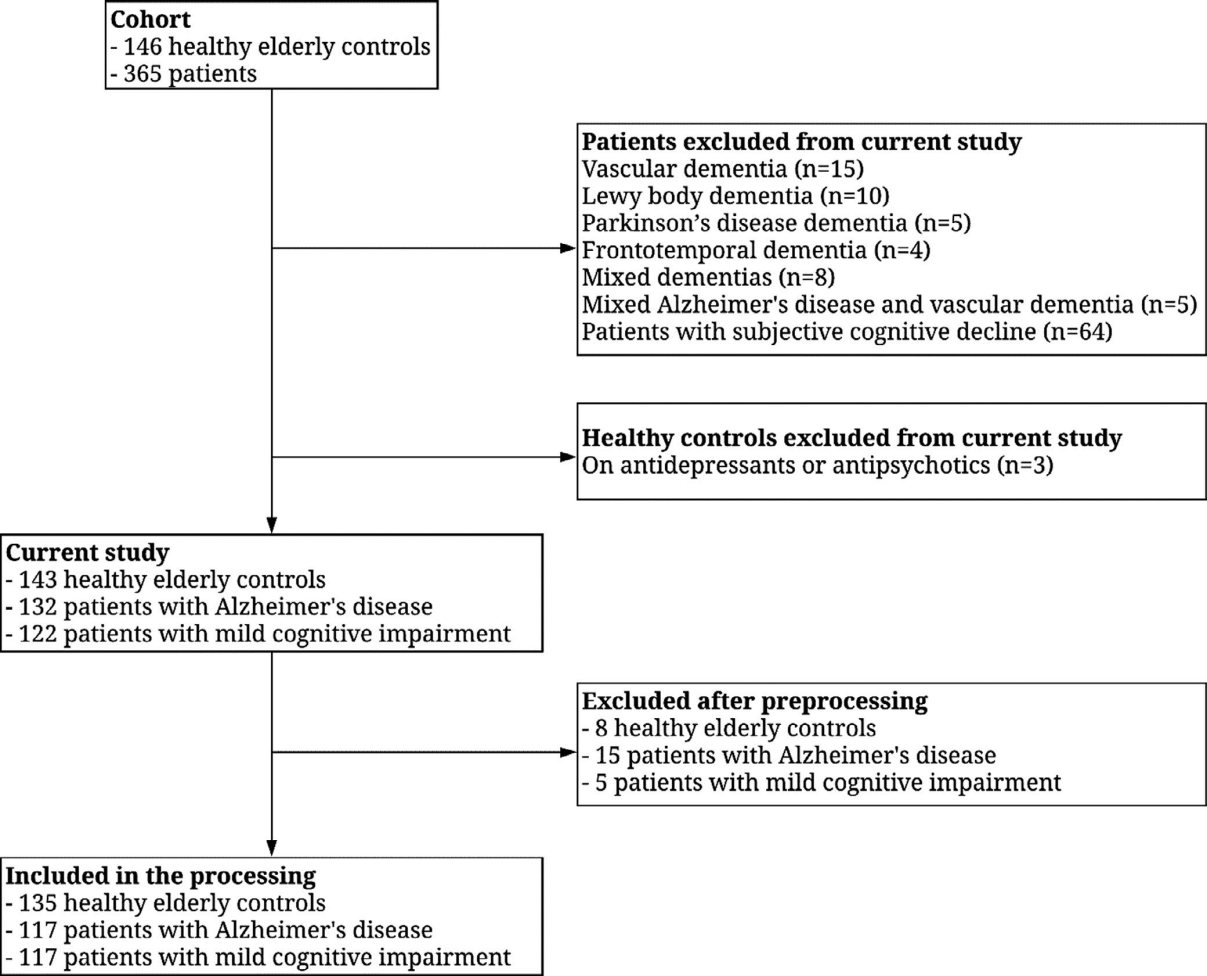
Low diagram of the number of included participants in the current study and of the excluded participants after preprocessing

The cohort was collected as part of a validation study where patients with other types of dementias were recruited (Engedal et al., 2015). In the current study, the following groups were excluded from analysis due to the low number of patients: vascular dementia (*n* = 15), Lewy body dementia (*n* = 10), Parkinson's disease dementia (*n* = 5), frontotemporal dementia (*n* = 4), mixed dementias (*n* = 8), and mixed AD and vascular dementia (*n* = 5). In addition, patients with subjective cognitive decline (*n* = 64) were also excluded since no follow‐up data on progression were available. Since some of the EEGs were of poor quality or lost, we had to exclude eight EEGs from HC, eight from patients with MCI, and 15 from patients with AD. In addition, three HC were excluded due to use of either antidepressants or antipsychotics, since the underlying condition and/or severity was unknown (see Figure 1). Table 1 presents a full description of the final sample.

**TABLE 1:** Characteristics of study participants

	HC	MCI	AD	*p*‐value
	(*n* = 135)	(*n* = 117)	(*n* = 117)	AD versus MCI versus HC
Mean age (*SD*), years	66.44 (7.64)	70.15 (8.13)	75.49 (7.65)	<.001
Sex (female), %	60.7	53	60.7	.377
Education, years (*SD*)	13.89 (3.61)	11.57 (3.92)	10.07 (3.39)	<.001
Antipsychotics	0	1	4	
Antidepressants	0	25	18	
Tranquilizers/hypnotics	3	12	6	
Antidementia drugs	0	2	18	
Painkillers	4	6	4	
Lumbar punctures performed		38	32	
Amyloid beta 42, mean (*SD*)		876.16 (354.92)	516.34 (114.52)	<.001
Total tau, mean (*SD*)		420.98 (230.51)	532.31 (273.85)	.069
Phosphorylated tau, mean (*SD*)		63.16 (26.72)	106.33 (114.52)	.027
MMSE, mean (*SD*)	28.91 (1.34)	27.11 (2.16)	23.52 (3.79)	<.001
Word list memory, mean (*SD*)	20.49 (3.99)	15.25 (4.29)	11.06 (3.83)	<.001
Word list recall, mean(*SD*)	7.39 (1.69)	3.66 (2.34)	1.52 (1.64)	<.001
Word list recognition, mean (*SD*)	19.47 (1.44)	17.92 (2.01)	15.84 (2.62)	<.001
Number of one‐second epochs	113.93 (55.49)	119.64 (48.23)	130.82 (53.14)	.038

Abbreviations: AD, Alzheimer's disease; HC, healthy controls; MCI, mild cognitive impairment; MMSE, mini‐mental state examination; *SD*, standard deviation. *p*‐values show the differences when comparing patients with AD, patients with MCI, and HC.

### 2.2 Clinical diagnostic assessment

All patients underwent a clinical diagnostic assessment comprised of (a) history from the patient and an informant; (b) physical examination focusing on neurological and cardiology status; (c) blood tests to screen for disorders that could be associated with cognitive impairment, such as B12 vitamin deficiency or low thyroid hormone levels; and (d) CT or MRI of the brain to evaluate white matter changes, general atrophy, and atrophy of the medial temporal lobes. Some of the patients underwent neuropsychological tests covering various cognitive domains, and some underwent a lumbar puncture to examine amyloid beta‐42, total tau, and phosphorylated tau protein in the cerebrospinal fluid, when indicated (see Table 1). Some patients were assessed with fluorodeoxyglucose PET or Tc‐HMPAO SPECT. A previously published paper contains the details of the clinical assessments (Braekhus, Ulstein, Ulstein, Wyller, & Engedal, 2011) outlined above.

The clinical diagnoses, which applied the Diagnostic and Statistical Manual of Mental Disorders, Fourth Edition, Text Revision, and the McKhann criteria, were made at consensus conferences at each memory clinic, or by at least two experienced physicians (McKhann et al., 1984). Winblad criteria were used to diagnose MCI (Winblad et al., 2004). All diagnoses were made blinded to the EEG results.

All HC were interviewed, and previous and present disorders and drug use were recorded. Any individual with a cognitive test score one standard deviation below the mean for age on the Mini‐Mental State Examination (MMSE), the clock‐drawing test (CDT), or the Consortium to Establish a Registry for Alzheimer's Disease (CERAD) score was excluded (see below).

### 2.3 Cognitive tests

For each patient, we conducted the MMSE (Engedal, Haugen, Haugen, Gilje, & Laake, 1988; Folstein, Folstein, Folstein, & McHugh, 1975), which is a measure of overall cognitive function consisting of 20 items with a minimum score of 0 and a maximum score of 30, with lower scores indicating more severe cognitive impairment; the clock‐drawing test (Shulman, 2000), which is a short screening test of cognition and visuospatial function with a minimum score of 0 and a maximum score of 5, with lower scores indicating poorer function, and the CERAD ten‐word list) (Morris, Mohs, Mohs, Rogers, Fillenbaum, & Heyman, 1988), which tests the ability to learn ten words with three repetitions (CERAD learning max. score = 30), to recall the ten words after ten minutes (CERAD recall max. score = 10), and to recognize the ten words among another ten different words (CERAD recognition max. score = 20). We used a modified version of the Montgomery Åsberg Depression Rating Scale to assess whether the patients had any depressive symptoms (Montgomery & Asberg, 1979). Each of the ten items was assessed as present or not, yielding a minimum score of 0 and a maximum score of 10.

The controls were tested with the MMSE, clock‐drawing test, and the CERAD ten‐word tests (all three parts), while depression was rated with the modified Montgomery Åsberg Depression Rating Scale in the same way as the patients.

### 2.4 EEG recording

The EEGs were recorded using a 19‐channel NicoletOne EEG System (Natus^®^). The sampling rate was different between the six sites ranging from 250 to 1,000 Hz. Electrodes were placed according to the international 10–20 system of electrode placement (Fp1, Fp2, F7, F3, Fz, F4, F8, T3, T5, T4, T6, C3, Cz, C4, P3, Pz, P4, O1, O2). Data were recorded using the average reference. Two bipolar electro‐oculography channels and one electrocardiogram channel were recorded to monitor artifacts. The EEGs were recorded, alternating between 3‐min periods each of eyes closed and eyes open, except for one center, where only continuous eyes‐closed segments were recorded. The participants were alerted if they became visibly drowsy.

### 2.5 Preprocessing EEG

The data were imported to MATLAB (Mathworks, v2016a) using the EEGLAB toolbox (Delorme & Makeig, 2004). The eyes‐closed segments were selected from the three‐minute, eyes‐open, and eyes‐closed periods. If the EEG only contained eyes closed, then the segments were selected from the first 10 min of the recording to prevent inclusion of segments with drowsiness or sleep. The electrodes were computationally located on the scalp by means of the DIPFIT toolbox (Oostenveld, Fries, Fries, Maris, & Schoffelen, 2011). The auxiliary channels (electrocardiogram and reference electrodes) were removed, and the data were bandpass‐filtered from 1–70 Hz and subsequently band‐stop filtered from 45–55 Hz using the *pop_firws* function in EEGLab with a filter order of two. The Kaiser window parameter beta was estimated using a maximum passband ripple of .001. The data were then down‐sampled to 200 Hz. Next, the data were divided into one‐second epochs that were visually inspected to remove epochs containing excessive noise or artifacts. Channels with excessive noise, drift, or reduced connection were rejected and interpolated using spherical interpolation. Recordings with four or more electrodes with excessive artifacts were excluded from the analysis. Afterward, the data were rereferenced to the average reference and independent component analysis was performed using the extended infomax algorithm (Lee, Girolami, Girolami, & Sejnowski, 1999), extracting up to nineteen components based on the data rank. This was done for each file, and components containing eye blinks, eye movement, ECG artifacts, or specific line‐noise artifacts were removed. Finally, the EEGs were visually inspected again, and epochs with excessive noise or artifacts were removed. The additional step of epoch removal was performed to assure that any artifacts, which could not be removed using independent component analysis, were removed. The investigator was blinded to diagnosis.

### 2.6 Microstate analysis

**FIGURE 2:**
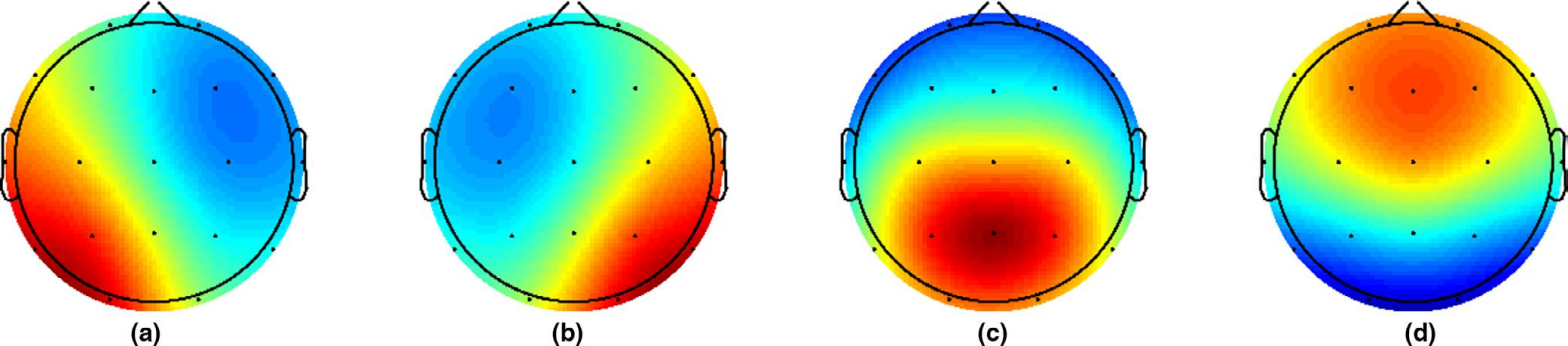
Global maps calculated based on the aggregated dataset from all participants and back‐fitted to each EEG recording. (A) to (D) assigned according to previous literature

**FIGURE 3:**
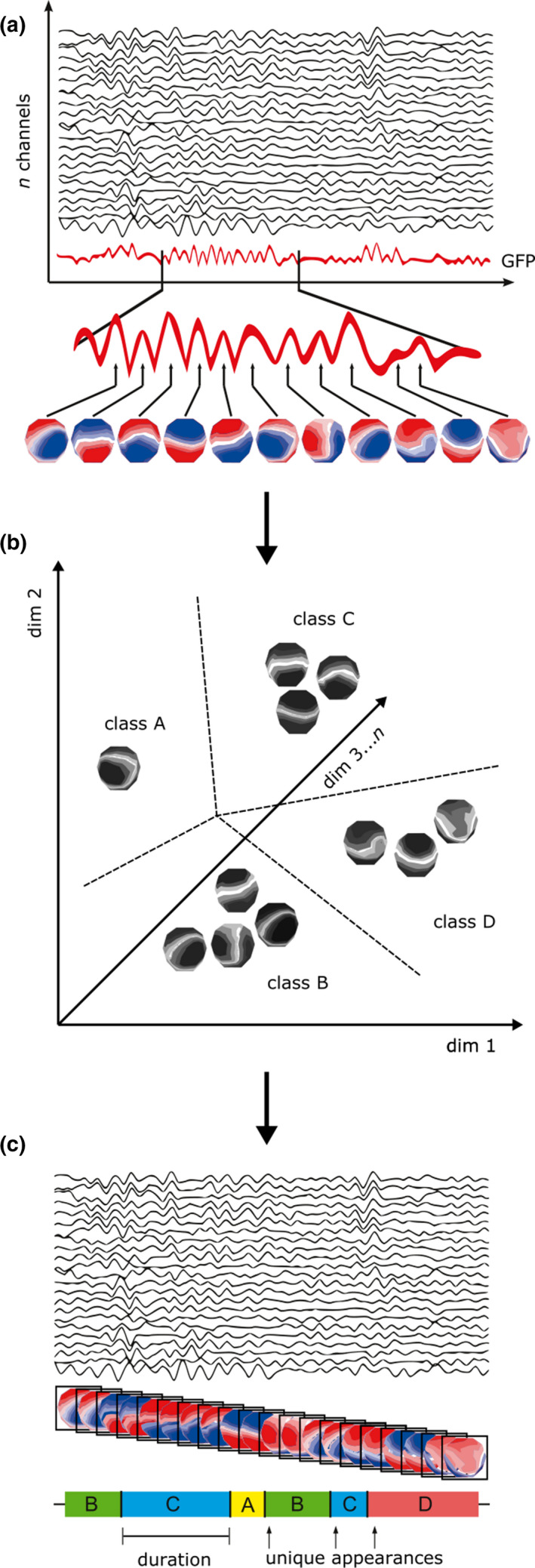
General schema for EEG microstate analysis. (a) The global field power (GFP) of the multichannel EEG signal is calculated at each time point. Local maxima of the GFP curve represent points of greatest topographic signal‐to‐noise ratio. These points are sampled and re‐expressed as a topography of electric potentials at each of the n electrodes in the electrode array. (b) These topographies are subjected to a modified k‐means clustering analysis. Each topography is plotted in n‐dimensional space, and the modified k‐means algorithm is applied to partition the resulting n‐dimensional space into optimal clusters. An arbitrary label, A, B, C, and D, is assigned to each cluster. (c) Each topography at local maxima of the GFP curve is assigned a label, A, B, C, or D, according to the cluster it belongs to. The multichannel EEG signal is re‐expressed as a sequence of alternating labels. From this data re‐representation, values such as the average microstate class duration, frequency of unique occurrences of microstates, and microstate transition probabilities can be calculated

Before performing the microstate analysis using the Microstate EEGlab Toolbox, we lowpass‐filtered the data at 40 Hz with the same settings as mentioned above (Poulsen, Pedroni, Langer, & Hansen, 2018). For each subject, we began by extracting the first 1,000 electric field montages at global field power (GFP) peaks with a minimum peak distance of 10 ms. GFP peaks that exceeded two times the standard deviation of the GFPs of all maps were excluded. To identify topographic clusters within these data, we submitted all *n* × 1,000 electric potential topographies to a modified K‐means clustering algorithm (Pascual‐Marqui, Michel, Michel, & Lehmann, 1995). Polarity of the EEG topography was ignored (Lehmann, 1971; Pascual‐Marqui et al., 1995; Wackermann, Lehmann, Lehmann, Michel, & Strik, 1993). We chose to predefine the sought number of microstates as four to remain consistent with the majority of prior studies of EEG microstates (Khanna et al., 2014) and because four have been confirmed to generate reproducible maps (Khanna, Pascual‐Leone, Pascual‐Leone, Michel, & Farzan, 2015). The number of repetitions was set at 50, while the maximum number of iterations was set at 1,000. To test whether the algorithm was affected by local minima issues, we repeated the entire analysis three times and found that the main findings can be robustly recovered (see Supplementary material, pp. 3–7; Figure S2; and Tables S1–S3). A set of four global maps was generated (see Figure 2) and back‐fitted to the whole EEG. To reduce noise, we rejected microstate segments shorter than 30 ms, which is the default in the toolbox (Poulsen et al., 2018). This was done to assure that the short segments, which may have been due to noise, did not affect the results. After back‐fitting the global maps, we calculated global explained variance (GEV), duration, occurrence, coverage, and the syntax for EEG files. Here, the duration is defined as the average time for each map to be present before transitioning to another map. Occurrence is defined as the average number of times a microstate occurred during the entire EEG. Coverage is defined as the total percent of the EEG for which a microstate was accounted for. GEV is defined as the variance of EEG activity explained by all four microstates. See Figure 3 for an overview of the microstate analysis pipeline.

However, if transitions from one state to the next occurred randomly, observed transition values would be proportional to the relative occurrence of the microstate classes. To test this, we performed syntax analyses based on the same analysis as previously described in detail (Lehmann et al., 2005; Nishida et al., 2013). We calculated the observed transitions based on all transitions, and then, the expected transitions based on the occurrence of the microstates for each subject separately. Afterward, these values were averaged across subjects for each group, and the difference was assessed using the chi‐square distance. To statistically test the difference, we performed a permutation test with 5,000 repetitions and randomly assigned the labels “expected” and “observed” to the subjects’ sets of twelve transition probabilities, after which the chi‐square distance was computed.

The majority of studies have used four microstates (Michel & Koenig, 2018). This type of parcellation of the dataset leaves a large part of the GEV unexplained, which is why we repeated the analyses described above for three, five, and six microstates (see Supplementary material, pp. 8–14).

**FIGURE 4:**
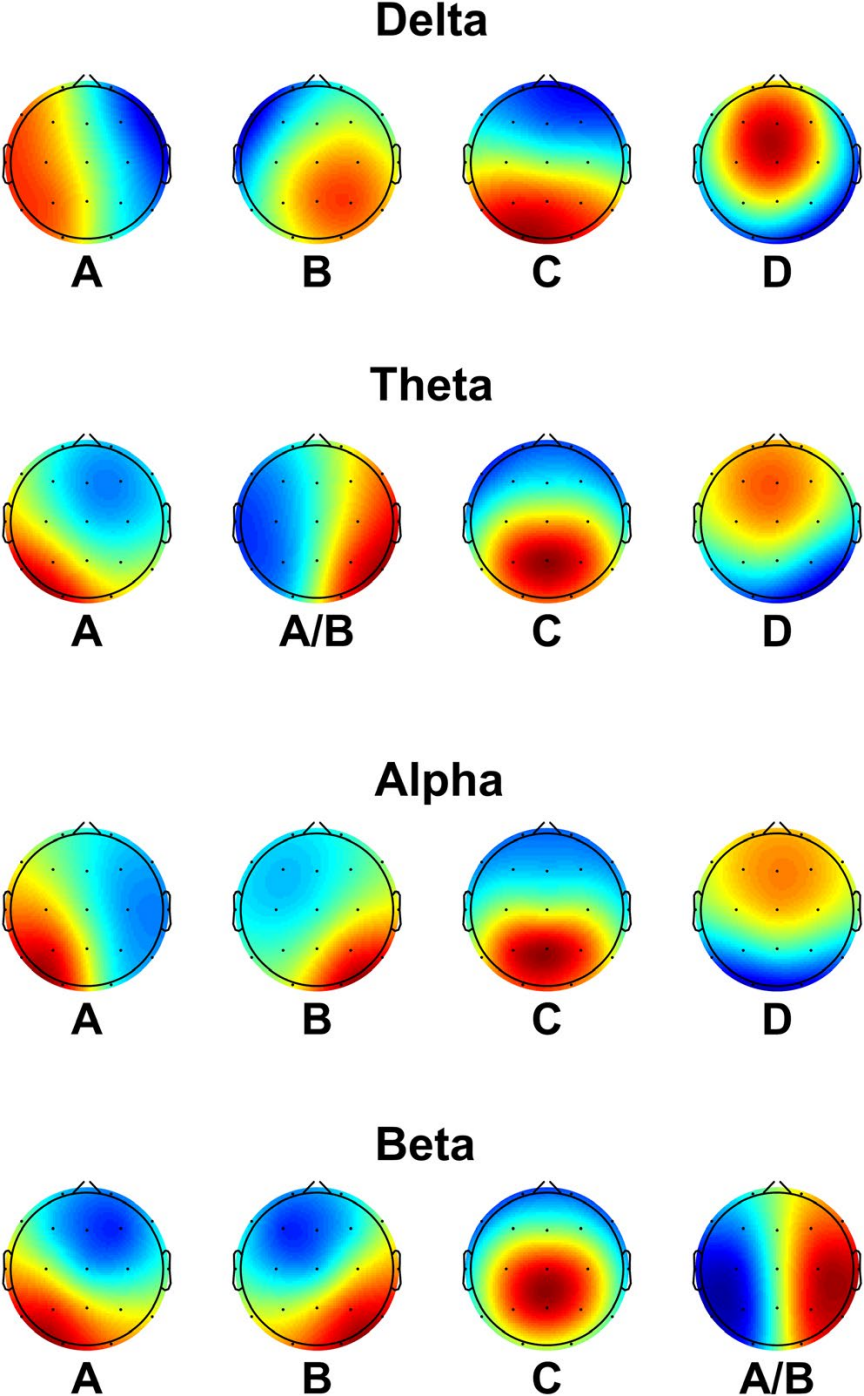
Global maps from the frequency‐specific microstates for delta (1–4 Hz), theta (4–8 Hz), alpha (8–13 Hz), and beta (13–30 Hz). The maps were calculated based on the aggregated dataset from all participants and back‐fitted to each EEG recording. (A) to (D) assigned according to previous literature

To investigate whether the microstate changes were located to specific frequency bands, we performed microstate calculations for the following frequency bands: delta (1–4 Hz), theta (4–8 Hz), alpha (8–13 Hz), and beta (13–30 Hz), see Figure 4 for global maps. For the delta band, we low‐pass filtered the data below 4 Hz, while bandpass filtering was performed for the other frequency bands using the same filter settings as mentioned above. For the subsequent microstate analysis, we performed the same analysis as mentioned above.

Finally, we noted that the average age of subjects in the HC cohort was less than that of the AD and MCI groups. To explore what effect this age difference may have had on microstate results, we conducted an additional analysis removing the 18 youngest HC subjects (thereby including 117 participants in each group) and repeated the microstate analysis as described above.

### 2.7 Statistics

All statistics were performed in MATLAB (vR2016a). To compare sex, we performed a chi‐square test. For number of one‐second epochs, age, years of education, the MMSE, and the ten‐word list of the CERAD score and subscores (learning, recognition and recall), we performed a one‐way ANOVA for the three groups.

When comparing the microstate values, we log‐transformed the data first due to the non‐normal distribution. For comparing AD, MCI, and HC, we performed an ANCOVA (Gruner, 2020) using age, sex, years of education, and current medications as covariates. Current medications were included as binary values for whether the person received antipsychotics, antidepressants, hypnotics, antidementia drugs, or painkillers. To correct for multiple comparisons, we performed false discovery rate (FDR) correction for 24 comparisons. Afterward, we computed post hoc tests using the same covariates as described above on the log‐transformed data if the group comparison was significant after FDR correction using a general linear model. The post hoc tests were considered significant at a *p* < .05. The same analyses were performed for the frequency‐specific microstates, and the correction for multiple comparisons was done for each frequency band separately.

For correlations, we chose to use the recall score and the learning score from the CERAD ten‐word list since neuropsychological studies have demonstrated that learning and recall best discriminate between HC and AD (Collie & Maruff, 2000; Twamley, Ropacki, Ropacki, & Bondi, 2006), with the recall score being the most sensitive in the early phases of AD (Welsh, Butters, Butters, Hughes, Mohs, & Heyman, 1991). We performed partial correlation using the same covariates as described above between duration, coverage, and occurrence (see definition under *Microstate analysis*) of microstate A and the recall and learning scores from the CERAD ten‐word list, including MMSE, amyloid, total tau, and phosphorylated tau. Afterward, we performed FDR correction for all *p*‐values. If the adjusted *p* < .05, it was considered significant.

### 2.8 Prediction

The dataset, which consisted of the z‐scores of duration, occurrence, and coverage for each microstate, as well as analysis of the microstate syntax (i.e., frequency of transitions among microstates), was compressed using principal component analysis such that 99% of the data variance was retained for the subsequent classification analysis. For the classification, we used multinomial regression for the three‐class classification of AD, MCI, and HC, implemented using the minFunc optimization procedure (Schmidt, 2005). Afterward, we used logistic regression for the two‐class classification, also implemented using the MATLAB code for logistic regression, which included HC and AD. We quantified the model using leave‐one‐out cross‐validation. The classification accuracies are reported averaged over the number of observations (i.e., subjects) left out one at a time in the leave‐one‐out cross‐validation procedure. This procedure was also performed using the results from the main analysis (1–40 Hz) and the results from the frequency‐specific microstates (24 values × 5 frequency bands). Furthermore, we used the same method using multinomial regression for the three‐class classification and logistic regression for the two‐class classification when removing the youngest 18 HC.

In addition, we performed linear discriminant analysis, quadratic discriminant analysis, and support vector machine for the three‐class classifications. Here, we did not perform principal component analysis when using linear discriminant analysis or support vector machine. The support vector machine for the three‐class problem was implemented using the default settings in MATLAB specified to use hyperparameter optimization of all parameters as defined in the code.

## 3 RESULTS

### 3.1 Demographics, cognitive tests, and AD biomarkers

Table 1 provides a full overview of demographics, cognitive test scores, AD biomarkers, and number of 1‐s epochs for the three groups: AD, MCI, and HC. We found significant difference in both age and education for patients with AD and MCI as compared with HC (*p* < .05).

### 3.2 Microstate results

There was no significant difference in the total GEV between the three diagnostic groups (*p* = .970, *F* = 0.031, *df* = 366), with an average GEV across groups of 49.51%.

We found a significant difference between AD, MCI, and HC for the duration of microstate A, with post hoc test using a general linear model showing a significant difference between AD and HC (*p* = .002, *t*‐value = 9.748, *df* = 250), and between MCI and HC (*p* = .004, *t*‐value = 8.633, *df* = 253). Furthermore, significant differences were found between AD, MCI, and HC for coverage of microstate A, with a significant difference between AD and HC (*p* < .001, *t*‐value = 15.111, *df* = 250), and between MCI and HC (*p* = .003, *t*‐value = 8.985, *df* = 250). We also found a significant difference in occurrence for microstate A, and the post hoc *t* tests showed significant difference between AD and HC (*p* < .001, *t* = 12.364, *df* = 250), and between MCI and HC (*p* = .012, *t* = 6.466, *df* = 250), see Table 2.

**FIGURE 5:**
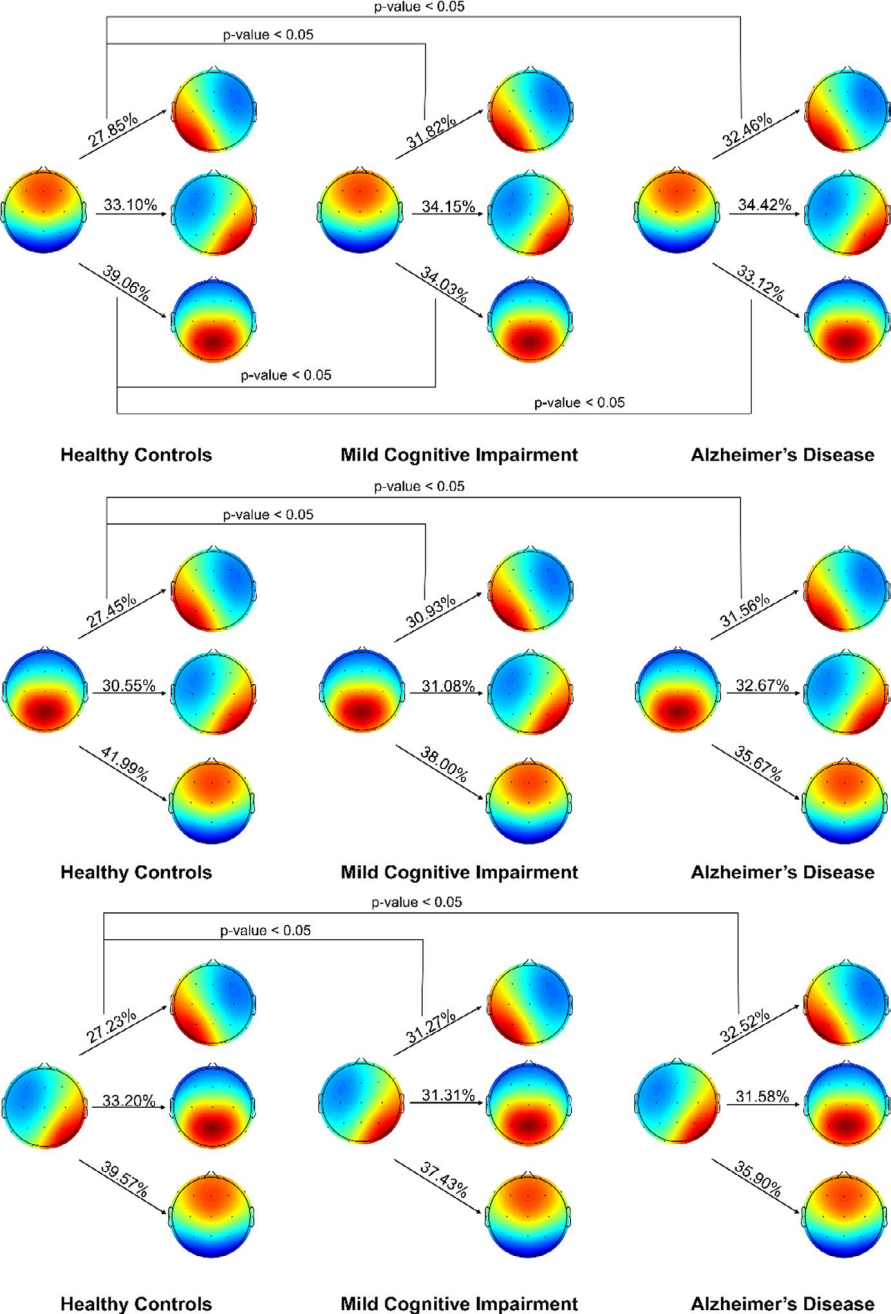
Significant results for the syntax analysis between healthy controls (HC), mild cognitive impairment (MCI), and Alzheimer's disease (AD). The first column is for HC, the second for MCI, and the third for AD. The values represent the percentage of times when for example microstates D transitioned to the other microstates. The figure shows that microstate B, C, and D were more likely to transition to microstate A in patients with AD and in patients with MCI

For the syntax analysis, we found a significant difference between the transition rate between AD, MCI, and HC from microstate D to C (*p*‐value_uncorr_ = .006, *p*‐value_corr_ = .024, *F* = 5.244, *df* = 357), D to A (*p*‐value_uncorr_ = .020, *p*‐value_corr_ = .002, *F* = 6.117, *df* = 357), C to A (*p*‐value_uncorr_ = .005, *p*‐value_corr_ = .024, *F* = 5.419, *df* = 357), and B to A (*p*‐value_uncorr_ = .012, *p*‐value_corr_ = .040, *F* = 4.500, *df* = 357), see Figure 5. However, no significant differences in the expected and observed transition probabilities were found for any of the groups (*p* > .05).

The microstate results for the comparisons between AD, MCI, and HC after removing the youngest 18 HC can be found in Supplementary material, pp. 15–16 (Figure S6 for global maps).

### 3.3 Frequency‐specific microstates

When investigating the frequency‐specific microstates, we found that both AD, and MCI showed significant increased duration and coverage of microstate A and decreased duration and coverage of microstate D in the delta band (*p* < .05). In the theta band, we found significantly increased duration of microstate A/B in patients and significantly decreased occurrence and coverage in microstate D (*p* < .05). Lastly, we found significantly decreased duration of microstate B and decreased occurrence and coverage of microstate A in the beta band. No significant differences were found in the alpha band. See Figure 4 and Table 3. When examining transitions, we found that B is significantly more likely to transition to A in AD as compared with both MCI and HC (*p* = .043, *F* = 6.233). No significant differences in the expected and observed transition probabilities were found for any of the groups (*p* > .05).

**TABLE 2:** Microstate features, including duration, occurrence, and coverage for microstates A‐D, and the FDR‐adjusted *p*‐value and *F*‐value for the comparison between AD, MCI, and HC. Degrees of freedom for all comparisons were 357

	Duration					Occurrence					Coverage				
	HC	MCI	AD	*p*‐value	*F*‐value	HC	MCI	AD	*p*‐value	*F*‐value	HC	MCI	AD	*p*‐value	*F*‐value
Microstate A, (*SD*)	75.32 (9.59)	80.30 (11.31)	81.47 (11.14)	.024	5.19	2.49 (0.69)	2.77 (0.70)	2.87 (0.61)	.020	6.26	19.01 (6.48)	22.64 (7.65)	23.62 (6.68)	.017	7.37
Microstate B, (*SD*)	79.15 (10.81)	82.02 (12.31)	82.53 (12.23)	.329	1.33	2.74 (0.63)	2.84 (0.61)	2.95 (0.55)	.307	1.48	21.88 (6.37)	23.53 (6.73)	24.61 (6.98)	.307	1.49
Microstate C, (*SD*)	85.67 (17.39)	83.58 (23.32)	81.68 (17.95)	.329	1.30	3.00 (0.67)	2.79 (0.67)	2.85 (0.68)	.163	2.59	26.17 (8.99)	23.81 (10.10)	23.83 (9.19)	.255	1.99
Microstate D, (*SD*)	101.88 (32.55)	95.93 (26.18)	90.83 (23.92)	.084	3.50	3.22 (0.51)	3.10 (0.49)	3.02 (0.55)	.513	0.71	32.94 (11.29)	30.02 (9.84)	27.95 (10.10)	.145	2.83

Abbreviations: AD, Alzheimer's disease; FDR, false discovery rage; HC, healthy controls; MCI, mild cognitive impairment; *SD*, standard deviation. *p*‐values show the differences when comparing patients with AD, patients with MCI, and HC.

### 3.4 Correlation

When performing partial correlation analysis adjusting for covariates, we did not find any significant correlation between the features of microstate A and the recall and learning scores from the CERAD ten‐word list, MMSE, amyloid, total tau, or phosphorylated tau when correcting for multiple comparisons using FDR. However, the largest rho obtained was between coverage of microstate A and recall from the CERAD (*p* = .018, *ρ* = −0.129). See Figure S1 for scatterplot of the correlation between coverage of microstate A and the recall score from the CERAD.

### 3.5 Prediction

For prediction analysis, we first used multinomial regression between AD, MCI, and HC and found an accuracy of 40.4% (sensitivity_MCI_ = 22.2%, sensitivity_AD_ = 38.5%, specificity = 57.8%). Since it was not possible for the classifier to distinguish well between MCI and the two other groups, we then compared HC and AD, where we found an accuracy of 62.7% (sensitivity = 57.3%, specificity = 67.4%) and HC and MCI, where we found an accuracy of 55.2% (sensitivity = 44.4%, specificity = 64.4%).

When applying linear discriminant analysis, we found almost the same accuracy between the three groups with an accuracy of 43.6% (sensitivity_MCI_ = 32.5%, sensitivity_AD_ = 35.9%, specificity = 60.0%). The same pattern was found using quadratic discriminant analysis for the three‐class classification with an accuracy of 39.8% (sensitivity_MCI_ = 12.8%, sensitivity_AD_ = 49.6%, specificity = 54.8%) and for support vector machine we found an accuracy of 41.2% (sensitivity_MCI_ = 18.8%, sensitivity_AD_ = 29.1%, specificity = 71.1%).

We also performed the multinomial regression using the results from the main analysis (1–40 Hz) and the results from the frequency‐specific microstates together. Here, we found an accuracy of 42.0% (sensitivity_MCI_ = 27.4%, sensitivity_AD_ = 37.6%, specificity = 58.5%) between all three groups. When using logistic regression, we found an accuracy of 69.8% (sensitivity = 68.4%, specificity = 71.1%) between HC and AD, and between HC and MCI, we found an accuracy of 58.7% (sensitivity = 52.1%, specificity = 64.4%).

For the analysis of the groups when removing the 18 youngest HC, we found an accuracy of 36.5% (sensitivity_MCI_ = 22.2%, sensitivity_AD_ = 36.8%, specificity = 50.4%). When investigating two classes, we found an accuracy of 62.8% (sensitivity = 66.7%, specificity = 59.0%) between AD and HC and an accuracy of 56.0% (sensitivity = 53.8%, specificity = 58.1%) between MCI and HC.

## 4 DISCUSSION

In the current study, we found significantly increased duration, occurrence, and coverage of microstate A in patients with AD and MCI compared to HC. When examining frequency‐specific microstates, we found that microstate A was affected in delta (1–4 Hz), theta (4–8 Hz), and beta (13–30 Hz), while microstate D was affected only in the delta and theta bands. Specific syntax alterations (the frequency of transitions from microstate D, C, and B to microstate A) in both patients with AD and MCI compared to HC were found, but no significant differences in expected or observed transition probabilities were found. Furthermore, we found a diagnostic accuracy between HC and AD of 69.8%. Together, we find that microstate features show poor diagnostic accuracy in patients with AD, but we find significant changes in microstate A features, which previously has been associated with temporal lobe function.

The majority of prior studies found shorter durations of all microstates in patients suffering from AD (Dierks et al., 1997; Stevens & Kircher, 1998; Strik et al., 1997), which has been suggested to indicate a temporal disorganization of global cortical networks in AD (Koenig et al., 2005; Nishida et al., 2013). However, these early studies used adaptive segmentation and did not group the microstates into specific classes (Dierks et al., 1997; Ihl et al., 1993; Stevens & Kircher, 1998; Strik et al., 1997), which may account for some of the differences between the present and earlier findings. A more recent study using cluster analysis (Nishida et al., 2013) separating the microstates into four clusters did not find any significant differences between patients with AD and HC but may have been underpowered. Another study using part of the same sample as described here found that microstate A was most affected in patients with AD and MCI compared to HC (Musaeus, Nielsen, et al., 2019). In the present study, overall microstate duration, occurrence, and coverage were either increased (microstates A and B) or decreased (microstates C and D) in AD and MCI compared to HC, suggesting aberrancy in the temporal dynamics of large‐scale cortical networks in patients with AD and MCI. When comparisons were made by individual microstate classes, only microstate A was significantly different (see Table 2). Furthermore, we also examined the microstates in specific frequency bands, which has to our knowledge not been done before in patients with AD. Here, we found that microstate A was specifically altered in delta (1–4 Hz), theta (4–8 Hz), and beta (13–30 Hz), while microstate D was affected only in the delta and theta bands and microstate B showed decreased duration in the beta band. To understand the connection between EEG microstates and the spatial changes, studies have used source localization and found that the main sources of microstate A were localized in the left temporal lobe (Brechet et al., 2019; Custo et al., 2017). Furthermore, studies have also explored the association between microstates and both the blood oxygen level‐dependent signal and resting‐state networks measured with fMRI (Britz, Van De Ville, & Michel, 2010; Musso, Brinkmeyer, Brinkmeyer, Mobascher, Warbrick, & Winterer, 2010; Yuan et al., 2012). One study associated microstate A with activations in the superior and middle temporal gyri (Britz et al., 2010). In patients with AD, other studies have found that the temporal areas, and especially the hippocampus, showed decreased activity during encoding of new information (Golby et al., 2005; Kato, Knopman, Knopman, & Liu, 2001; Machulda et al., 2003). This supports that microstate A is associated with areas of the brain that has been shown to be affected in patients with AD. This difference in affected side has previously been reported in MR studies, which showed that atrophy was more pronounced on the left side (Baron et al., 2001; Killiany et al., 2000) in patients with AD. Furthermore, the studies using source localization showed that microstate A corresponds to the left temporal lobe (Brechet et al., 2019; Custo et al., 2017). The reason for only finding significant differences in the left side may be due unintentional selection bias toward patients referred with language affection or evidence that early changes in perfusion as measured with SPECT in AD are more prominent on the left side (Hogh, Madsen Sjo, Madsen Sjo, Gade, & Waldemar, 2004). Alternatively, patients with right‐hemisphere dominant AD may be more likely to lack insight into symptoms, possibly delaying presentation and further contributing to referral selection bias. The underlying reason for the frequency‐specific changes could be associated with the changes in spectral power. Here, we found that especially the theta band is affected in the early stage of the disease (Musaeus, Engedal, et al., 2018). Due to short EEG recordings, we did not investigate any potential topographical changes. Future studies should include longer EEG recordings to investigate whether any topographical changes exist. When testing whether there were any differences in expected and observed transition probabilities, no significant changes were found. Since microstate A is associated with the temporal lobe, the changes may be related to the neuropathological findings in AD as described by the Braak stages (Braak & Braak, 1991; Thal, Rub, Rub, Orantes, & Braak, 2002), which are especially pronounced in the temporal lobes in early AD. Furthermore, follow‐up studies using PiB‐PET, which quantifies the beta‐amyloid deposition, have found that the temporal lobes are one of the first parts of the brain with beta‐amyloid deposition (Okello et al., 2009; Villemagne et al., 2011). Here, we hypothesize that the changes in microstate A may reflect the underlying pathological changes in the left temporal lobe.

**TABLE 3:** Microstate features, including duration, occurrence, and coverage for microstates A‐D, and the FDR‐adjusted *p*‐value and *F*‐value for the comparison between AD, MCI, and HC when investigating frequency‐specific microstates

	Duration					Occurrence					Coverage				
	HC	MCI	AD	*p*‐value	*F*‐value	HC	MCI	AD	*p*‐value	*F*‐value	HC	MCI	AD	*p*‐value	*F*‐value
Delta (1–4 Hz)															
Microstate A, (*SD*)	93.55 (13.05)	102.43 (15.72)	103.03 (16.31)	.041*	5.71	2.19 (0.43)	2.43 (0.43)	2.44 (0.38)	.072	4.25	20.99 (6.51)	25.45 (7.47)	25.61 (7.46)	.041*	5.35
Microstate B, (*SD*)	100.02 (10.18)	98.79 (9.82)	98.12 (10.41)	.916	0.20	2.44 (0.27)	2.41 (0.28)	2.39 (0.31)	.857	0.45	24.57 (4.61)	24.01 (4.50)	23.62 (4.94)	.857	0.39
Microstate C, (*SD*)	107.54 (11.91)	105.31 (11.54)	105.54 (12.99)	.857	0.34	2.59 (0.33)	2.53 (0.35)	2.50 (0.35)	.857	0.41	28.13 (6.15)	26.95 (6.29)	26.68 (6.40)	.919	0.13
Microstate D, (*SD*)	101.94 (9.79)	96.19 (10.26)	97.70 (12.49)	.030*	6.80	2.56 (0.30)	2.43 (0.35)	2.43 (0.39)	.134	2.91	26.30 (5.00)	23.59 (5.37)	24.09 (6.49)	.046*	4.95
Theta (4–8 Hz)															
Microstate A, (*SD*)	111.92 (17.37)	120.41 (21.49)	120.25 (24.57)	.054	4.30	2.16 (0.34)	2.11 (0.37)	2.11 (0.38)	.611	0.61	24.31 (5.46)	25.43 (6.33)	25.56 (7.12)	.588	0.94
Microstate A/B, (*SD*)	102.53 (18.25)	115.18 (22.04)	120.67 (32.29)	.005*	8.65	1.92 (0.48)	1.95 (0.46)	2.04 (0.48)	.397	1.40	20.00 (7.14)	22.77 (7.95)	25.08 (9.34)	.054	4.21
Microstate C, (*SD*)	102.74 (13.93)	104.97 (20.34)	103.50 (21.24)	.611	0.58	2.30 (0.50)	2.09 (0.55)	2.07 (0.51)	.203	2.22	23.84 (6.82)	22.41 (8.29)	21.73 (7.60)	.611	0.59
Microstate D, (*SD*)	132.68 (33.64)	135.05 (49.43)	127.28 (30.99)	.099	3.24	2.39 (0.32)	2.18 (0.36)	2.14 (0.37)	.006*	7.75	31.86 (9.15)	29.39 (9.67)	27.64 (9.40)	.028*	5.76
Alpha (8–13 Hz)															
Microstate A, (*SD*)	157.38 (30.13)	169.29 (34.85)	169.74 (35.01)	.255	2.78	1.23 (0.42)	1.39 (0.39)	1.44 (0.42)	.181	3.60	20.00 (9.03)	24.14 (9.74)	25.24 (10.17)	.181	3.94
Microstate B, (*SD*)	176.28 (36.90)	170.85 (35.34)	169.36 (44.32)	.793	0.46	1.39 (0.33)	1.44 (0.31)	1.45 (0.31)	.793	0.37	24.66 (7.74)	24.92 (7.94)	24.89 (8.96)	.996	0.03
Microstate C, (*SD*)	196.86 (95.11)	184.15 (88.59)	177.39 (91.27)	.475	1.53	1.29 (0.31)	1.26 (0.30)	1.33 (0.33)	.340	2.07	25.57 (12.74)	23.67 (12.69)	24.18 (12.98)	.571	0.97
Microstate D, (*SD*)	210.02 (85.30)	190.88 (69.84)	184.09 (92.05)	.504	1.32	1.42 (0.28)	1.42 (0.26)	1.38 (0.31)	.996	0.00	29.77 (12.24)	27.28 (10.68)	25.69 (12.31)	.637	0.80
Beta (13–30 Hz)															
Microstate A, (*SD*)	91.25 (10.51)	89.19 (10.80)	86.77 (9.55)	.089	3.55	3.20 (0.32)	3.19 (0.34)	3.06 (0.41)	.043*	5.02	29.35 (5.35)	28.62 (5.74)	26.83 (6.00)	.043*	5.08
Microstate B, (*SD*)	91.42 (9.49)	87.21 (8.57)	87.38 (9.53)	.043*	5.28	3.18 (0.38)	3.13 (0.34)	3.11 (0.37)	.584	0.63	29.31 (5.75)	27.50 (4.95)	27.40 (5.85)	.154	2.62
Microstate C, (*SD*)	78.27 (8.74)	79.10 (12.73)	80.17 (11.17)	.348	1.24	2.44 (0.53)	2.49 (0.59)	2.58 (0.59)	.174	2.37	19.42 (5.82)	20.25 (8.16)	21.22 (7.63)	.189	2.15
Microstate A/B, (*SD*)	81.23 (10.26)	82.38 (9.67)	83.75 (10.37)	.556	0.72	2.65 (0.50)	2.82 (0.53)	2.88 (0.53)	.125	3.09	21.93 (6.66)	23.63 (6.72)	24.55 (7.05)	.183	2.25

Abbreviations: AD, Alzheimer's disease; FDR, false discovery rage; HC, healthy controls; MCI, mild cognitive impairment; *SD, *standard deviation. *p*‐values show the differences when comparing patients with AD, patients with MCI, and HC.

We also examined whether microstates could be used as a potential classifier of disease and found that microstate features are not satisfactory available to distinguish between three groups (AD, MCI, and HC), which probably is due to nonsignificant differences between AD and MCI. In fact, many patients with MCI may have AD at a subclinical dementia stage, which is supported by the notion that over 50% of the included patients with MCI progressed (“converted”) to AD within two years follow‐up in the Danish substudy (Musaeus, Nielsen, Osterbye, & Hogh, 2018). When comparing HC with AD, we found a poor classification rate of 69.8%. This rate is lower than between AD and HC using EEG spectral power (Musaeus, Engedal, et al., 2018) and lower than the discriminatory power of EEG connectivity (Musaeus, Engedal, Hogh, et al., 2019). The underlying reason could be that the changes in EEG microstates may not be present before later in the disease stages. The EEG segments were also too short to obtain four optimal maps if each participant's EEG was segmented as previously described (Koenig et al., 1999). We suggest that by recording longer EEGs and using segmentation for each person individually, it may be possible to increase the diagnostic accuracy.

We chose to extract four microstates since they are the most commonly reported ones and have been shown to be reliable (Khanna et al., 2014). We found that the algorithm was robust, and the main findings could be replicated (see Supplementary material, pp. 3–7). When looking at three, five, and six microstates, we found that patients with AD and MCI had a global affection of microstates, but there was a significant affection of microstate A when extracting both three, five, and six microstates. The results suggest that changes in microstate A are the hallmark of EEG microstate changes in AD. In addition, the accuracy did not differ markedly from the main analysis, which may be due to microstate A being the most important microstate in patients with AD. Furthermore, even though GEV was not significantly different between the three groups, it was low (average GEV = 49.51%) compared to what other studies have reported (normally reporting a GEV of >70% (Michel & Koenig, 2018)). The increase in GEV was minimal when increasing the number of microstates, and this difference may be due to the fact that previous studies recorded EEGs on younger participants. In the current analysis, we only included the first 534 GFP peaks in the segmentation to equalize contributions from longer EEG files and this may also lead to a lower GEV. In addition, the low number of GFP peaks also limits our ability to investigate topographical differences in maps between groups. Furthermore, in the current study we had only 19 channels, which is below the number of channels used in recent microstate studies (Michel & Koenig, 2018). As a result, future studies should include longer EEG recordings to better determine whether any topographical changes exist.

### 4.1 Limitations of the study and future directions

The current study has some limitations. Cerebrospinal fluid markers were available for only 30% of the included patients, which limited the statistical power of the correlations between microstate features and cerebrospinal fluid markers. The lack of follow‐up data in the MCI group prevented us from investigating which patients with MCI converted to AD, but we hypothesize that most of them would ultimately develop AD, as supported the substudy mentioned above where 50% of the MCI progressed to AD (Musaeus, Nielsen, et al., 2018). Demographically, we found that the HC were younger than the AD and MCI patients, which impacts microstate features (Koenig et al., 2002). When removing the 18 youngest participants in the HC and thereby having a total of 117 participants in all three groups, we found similar results. A large proportion of AD and MCI patients were treated with medications (34.62%) that may have impacted the EEG, as seen in patients with schizophrenia (Merrin, Meek, Meek, Floyd, & Callaway, 1990). Furthermore, the HC group had a higher level of education than both clinical groups, which may also have played in assessing memory function. However, we tried to correct for these confounders by including age, use of drugs, and educational level as covariates when performing ANCOVA. Future studies investigating the changes in EEG microstates in patients with AD should strive to do longer EEG recordings in an effort to make it possible to extract individual maps for each participant. We hypothesize that this will increase GEV for each of the participants and may make EEG microstates an applicable diagnostic tool since differences between the diagnostic groups become clearer. Moreover, there was a significant difference in the number of one‐second epochs per subject, which may have affected our findings. In addition, future studies should investigate the relationship between EEG microstates and changes in metabolism as measured with FDG‐PET in patients with AD.

## 5 CONCLUSION

In the present study, we found evidence of EEG microstate changes in patients with AD. Specifically, there was a longer duration, larger coverage, and higher frequency of occurrence of microstate A among AD and MCI patients compared to HC. Furthermore, we found evidence that the microstates were due to microstate changes in specific frequency bands. This microstate has previously been associated with activity in the left temporal lobe using source localization and changes in the blood oxygen level‐dependent signal in the left temporal region, which is strongly affected by amyloid and tau pathology in patients with AD. Together, our results show that EEG microstate analysis may be a useful tool in examining dynamic network activity and disruption in AD. Future studies should examine the relationship between hypometabolism with FDG‐PET and the microstate features to understand the applicability of EEG microstates as a diagnostic tool.

## CONFLICTS OF INTEREST

None.

## AUTHORS' CONTRIBUTIONS

K.E., P.H., V.J., M.N., A.O., J.S., L.W., and B.A. initiated the study, recruited patients, and gathered patient data. C.M., M.M., and A.K. analyzed the data. C.M. wrote the first draft of the article. C.M., K.E., P.H., V.J., A.K., T.K., M.M., M.N., A.O., E.S., J.S., L.W., G.W., and B.A. have edited and revised the manuscript, and all authors have approved the final version of the manuscript.

## Supporting information

FigS1

FigS2

FigS3

FigS4

FigS5

FigS6

Supplementary Material

## Data Availability

The data that support the findings of this study are available from the corresponding author upon reasonable request. However, due to regulations, we are not able to share the EEG files.
