# Supplementary figures and images for: Changes in the left temporal microstate are a sign of cognitive decline in patients with Alzheimer’s disease
Source: Brain Behav. 2020 Apr 27;10(6):e01630. doi: 10.1002/brb3.1630 (PMC7303403; doi:10.1002/brb3.1630)

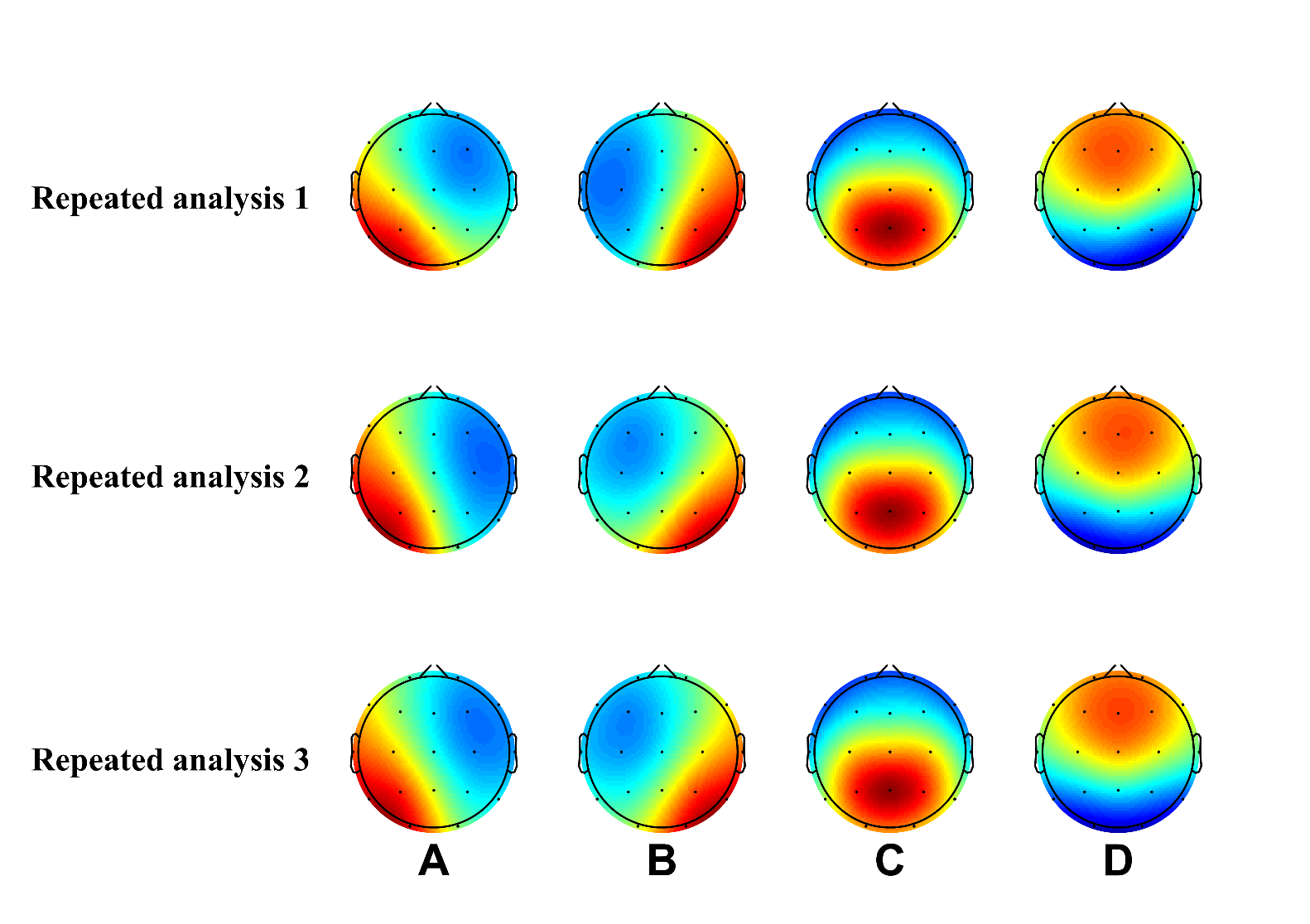

Supplement: Supplementary file 2 — FigS2 [file BRB3-10-e01630-s002.tif]

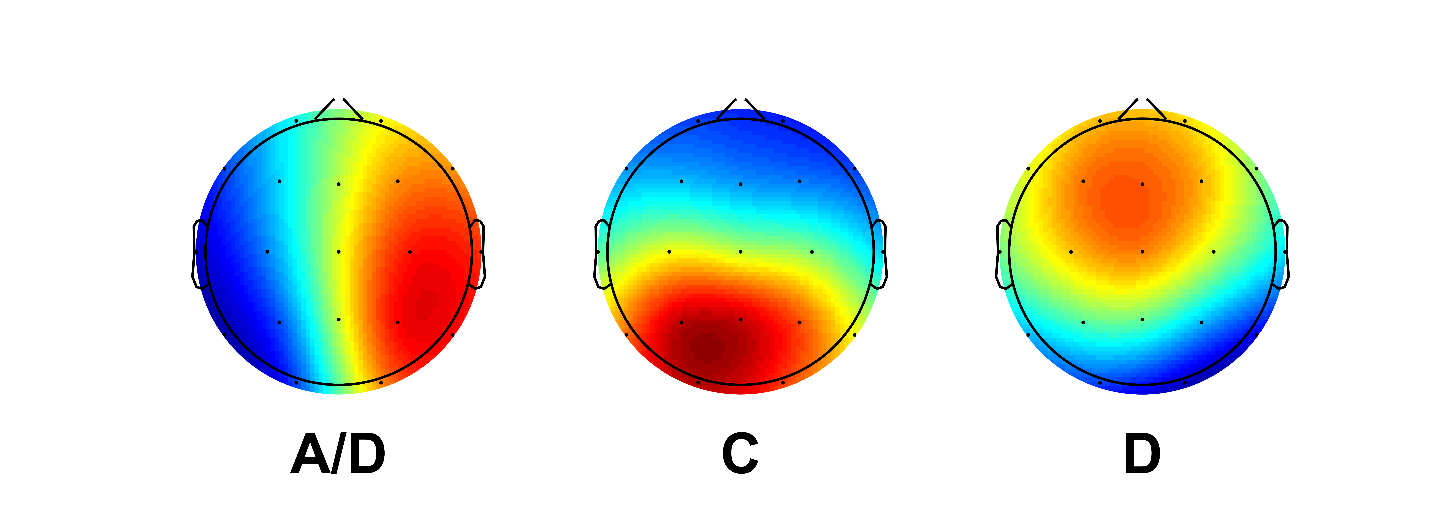

Supplement: Supplementary file 3 — FigS3 [file BRB3-10-e01630-s003.tif]

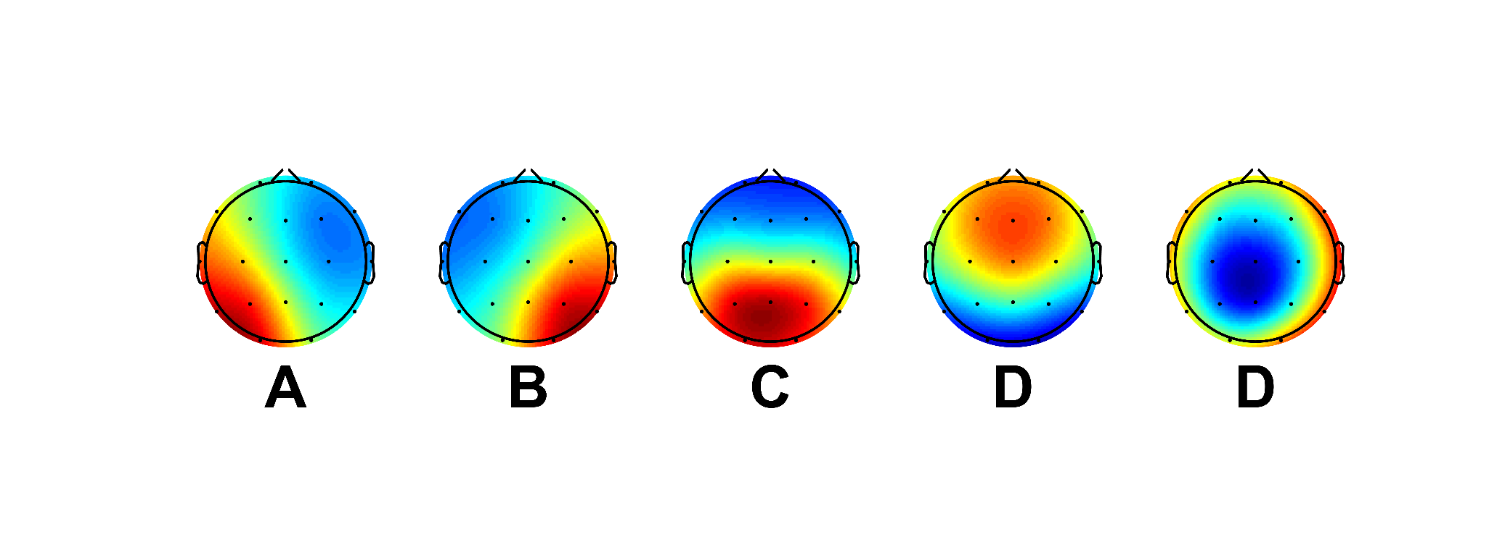

Supplement: Supplementary file 4 — FigS4 [file BRB3-10-e01630-s004.tif]

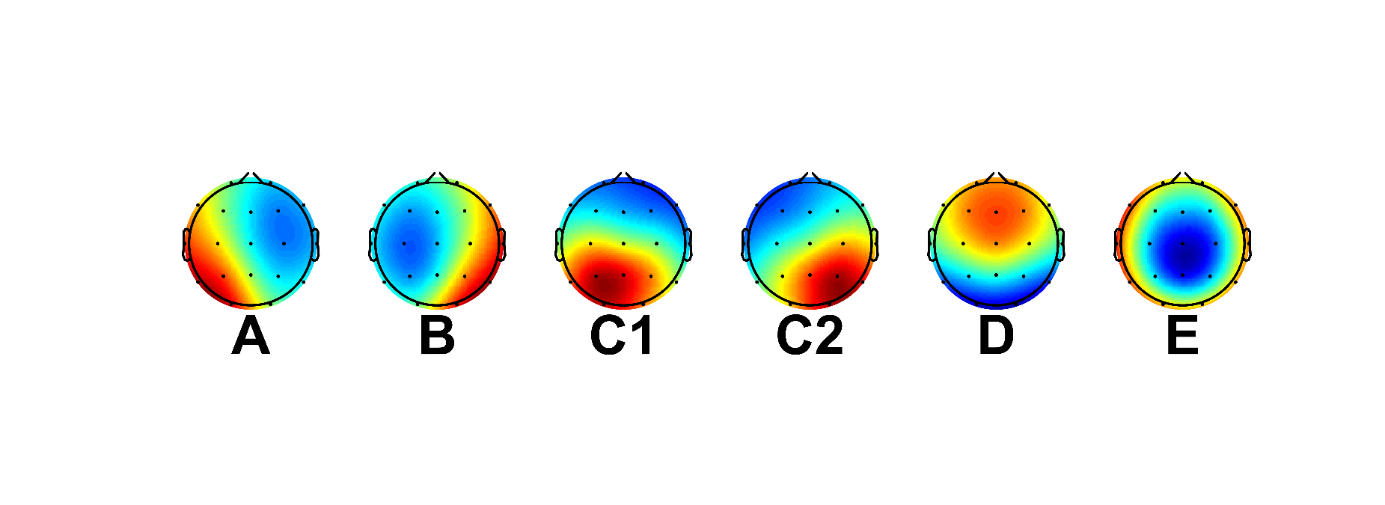

Supplement: Supplementary file 5 — FigS5 [file BRB3-10-e01630-s005.tif]

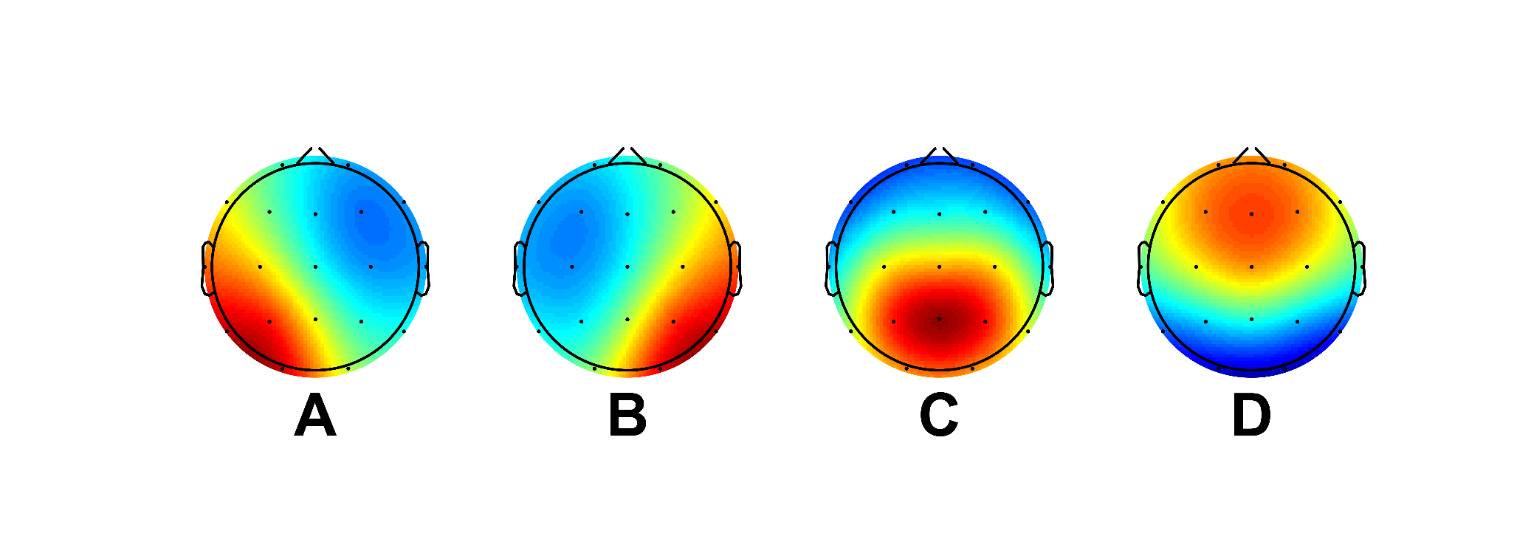

Supplement: Supplementary file 6 — FigS6 [file BRB3-10-e01630-s006.tif]
