# Supplementary material for: Changes in the left temporal microstate are a sign of cognitive decline in patients with Alzheimer’s disease
Source: Brain Behav. 2020 Apr 27;10(6):e01630. doi: 10.1002/brb3.1630 (PMC7303403; doi:10.1002/brb3.1630)
Supplement: Supplementary file 7 — Supplementary Material [file BRB3-10-e01630-s007.docx]

**Supplementary material**

Figure showing the scatterplots of the correlation between microstate A and the recall score of the CERAD, see page 2.

Repeating the analysis to test whether it is robust, see page 3-7

Results from three, five, and six microstates, see page 8-14

Results after removing the youngest 18 participants in the HC group, see page 15-16


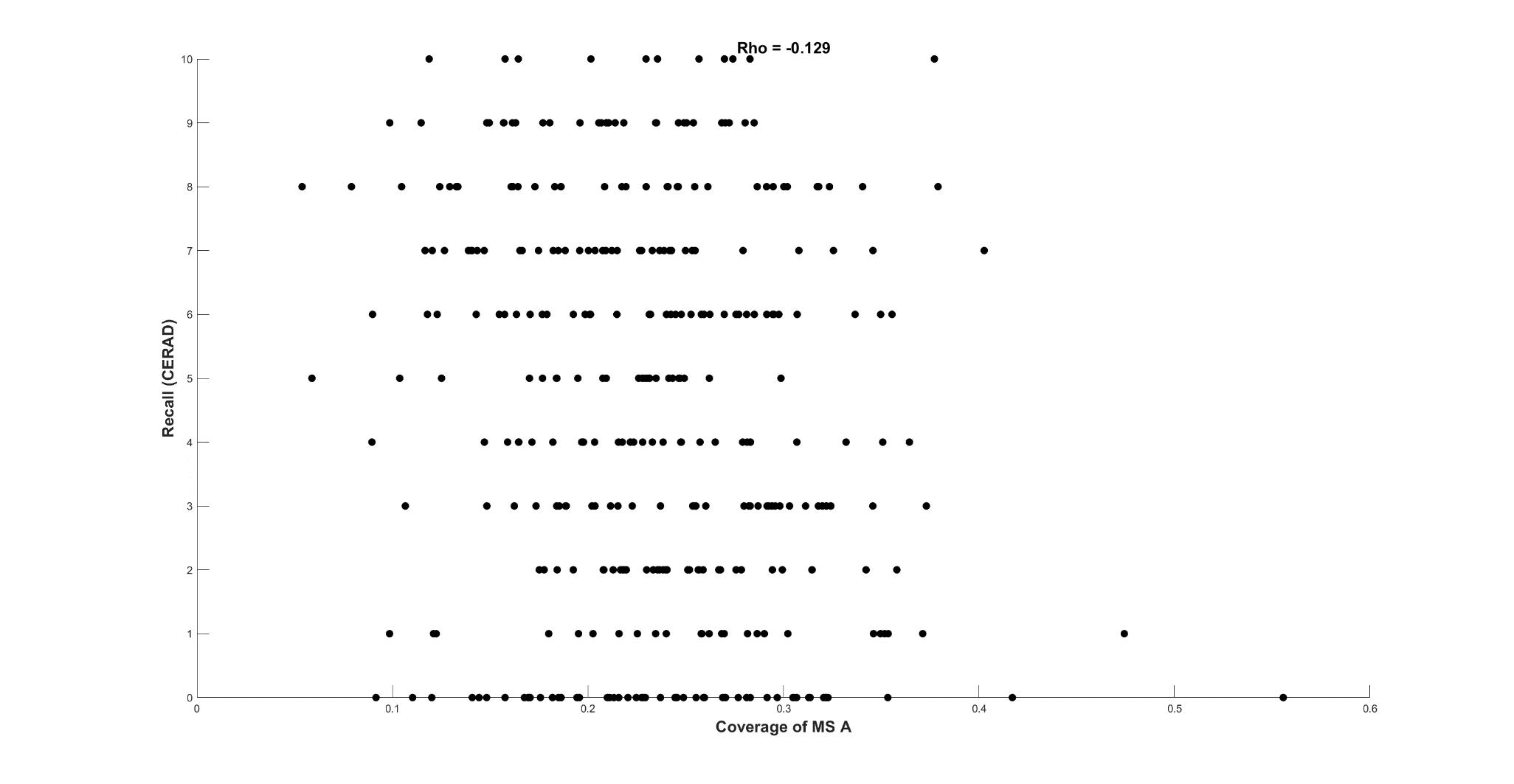


**Supplemental figure 1**: Scatterplots of the correlations between coverage and recall from the 10 word-list of the CERAD.

**Repeating the analysis to test whether it is robust**

To test whether the calculations were robust, we repeated the entire analysis three times, see below.


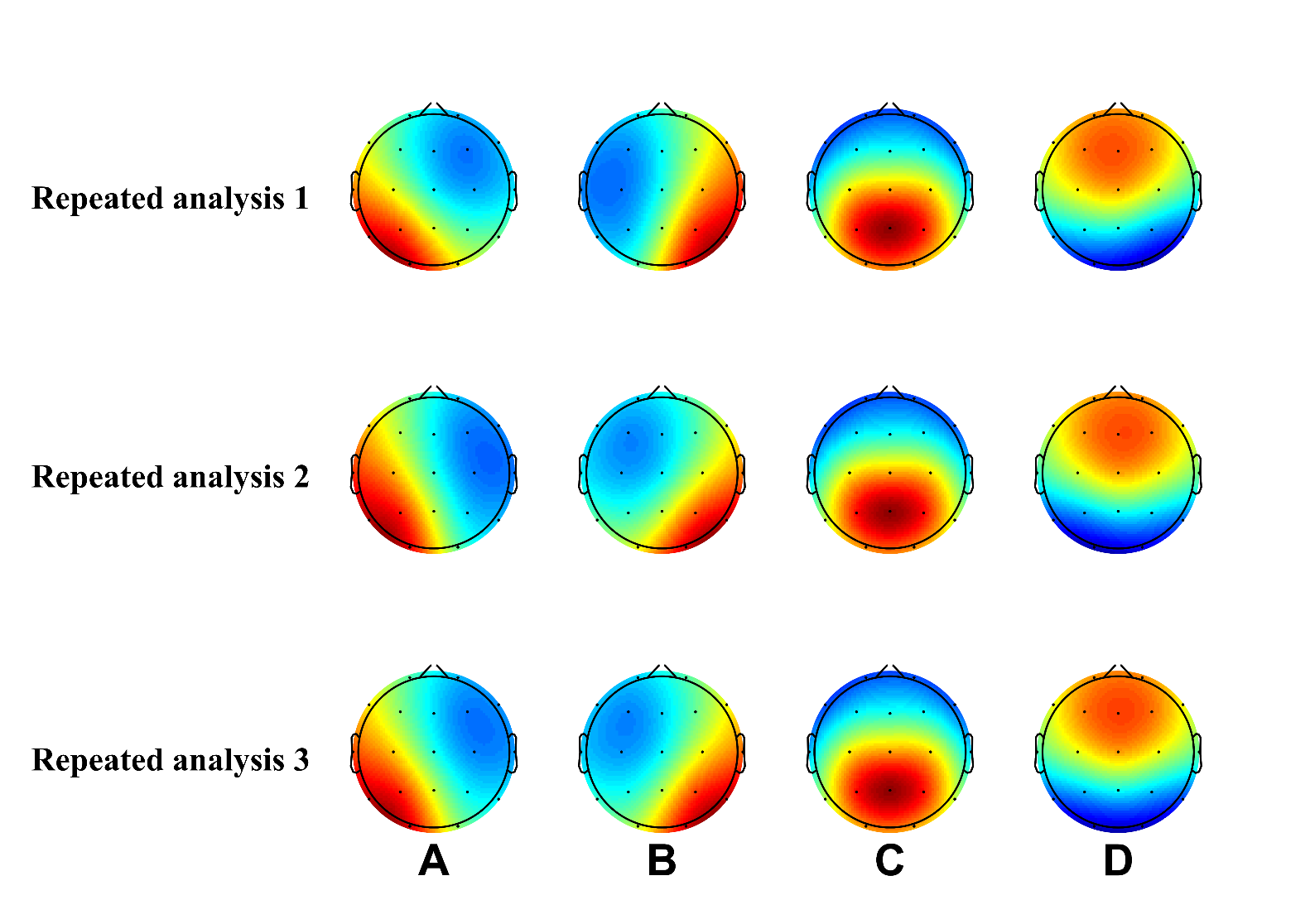


**Supplemental figure 2**: Overview of the maps from the three repeated analyses.

*Repeated analysis 1*

**Supplemental table 1**
Table showing the features, which include duration, occurrence, and coverage for each microstate (A-D) and the FDR adjusted p-value and F-value for the comparison between AD, MCI, and HC for the repeated analysis 1.

HC = healthy controls; AD = Alzheimer’s disease; MCI = mild cognitive impairment; SD is in brackets; MMSE = Mini-Mental State Examination.

*Repeated analysis 2*

**Supplemental table 2**
Table showing the features, which include duration, occurrence, and coverage for each microstate (A-D) and the FDR adjusted p-value and F-value for the comparison between AD, MCI, and HC for the repeated analysis 2.

HC = healthy controls; AD = Alzheimer’s disease; MCI = mild cognitive impairment; SD is in brackets; MMSE = Mini-Mental State Examination.

*Repeated analysis 3*

**Supplemental table 3**
Table showing the features, which include duration, occurrence, and coverage for each microstate (A-D) and the FDR adjusted p-value and F-value for the comparison between AD, MCI, and HC for the repeated analysis 3.

HC = healthy controls; AD = Alzheimer’s disease; MCI = mild cognitive impairment; SD is in brackets; MMSE = Mini-Mental State Examination.

**Conclusion**

Here, we found that the even though the values varied slightly between the different analyses, the general trends in the data were the same with significant differences for microstate A features.

**Results from three, five, and six microstates**

We used the same setting as described for four microstates except for changing the number of extracted microstates. Here, set of three (see Supplemental figure 3), five (see Supplemental figure 4), and six (see Supplemental figure 5) global maps were generated and back-fitted to each of the EEG files by labeling each GFP local maximum according to the global map it most closely correlated to.

1. *Results from extracting three microstates*

**
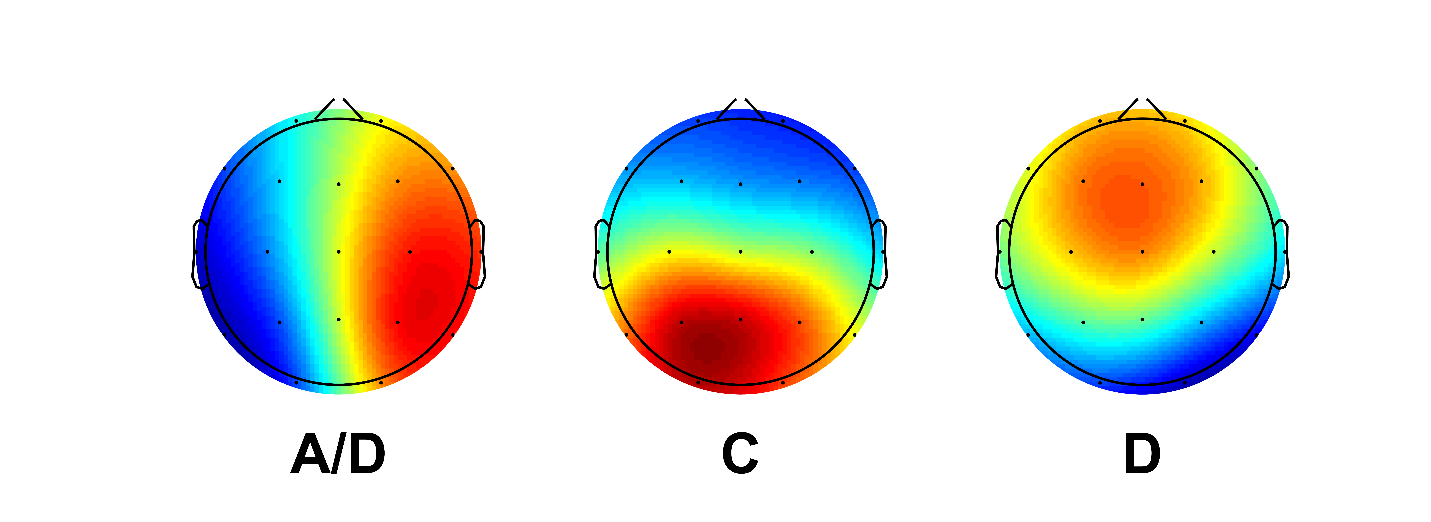
**

**Supplemental figure 3**: The global maps that were calculated based the aggregated dataset from all participants and were back-fitted to each of the EEG recordings. Here, we assumed that microstate A, and B were concatenated to A/B.

There was no significant difference in the total GEV between the three diagnostic groups (p-value = 0.833, F-value = 0.183) with an average GEV across groups of 47.58%.

*Prediction analysis*: We used multinomial regression for the three-class classification. Here, we found an accuracy of 40.4% (sensitivity_MCI_ = 8.5%, sensitivity_AD_ = 39.3%, specificity = 68.9%).

1. *Results from extracting five microstates*

**
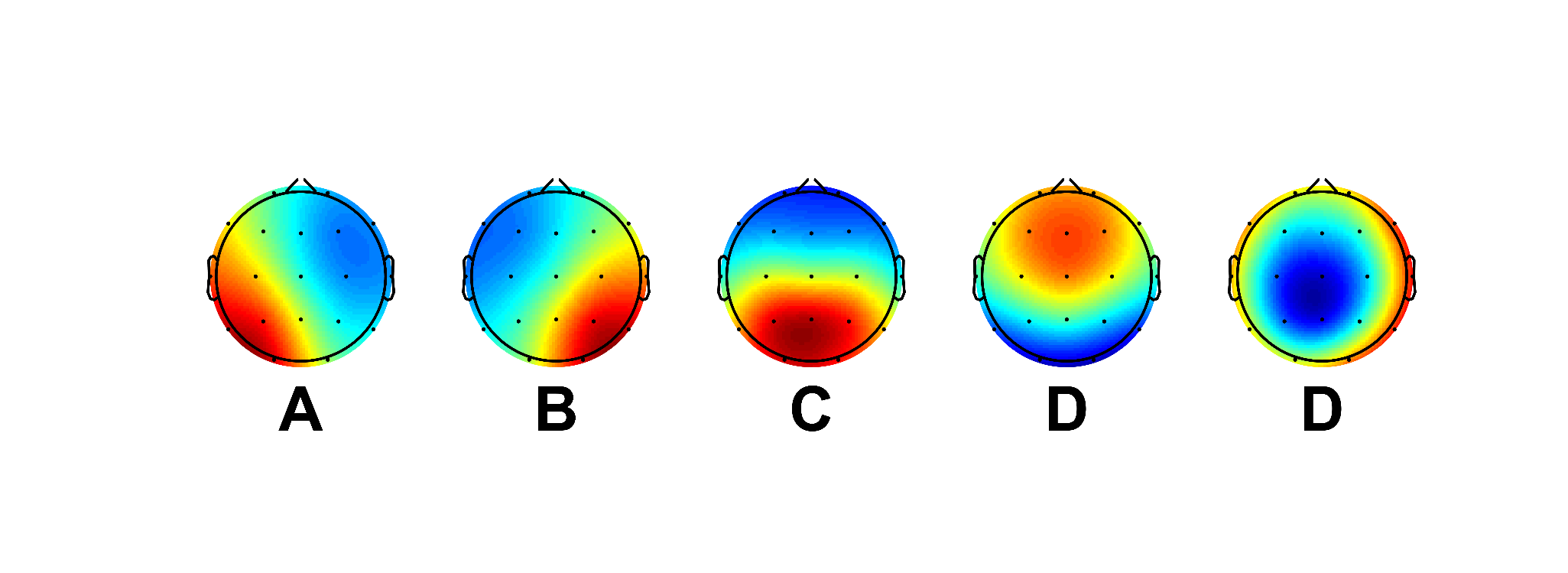
**

**Supplemental figure 4**: The global maps that were calculated based the aggregated dataset from all participants and were back-fitted to each of the EEG recordings. The first four microstates are labelled as A-D according to the literature in the field, and we labelled the fifth one as E.

There was no significant difference in the total GEV between the three diagnostic groups (p-value = 0.944, F-value = 0.058) with an average GEV across groups of 50.78%.

*Prediction analysis*: We compared HC and the disease groups AD, and MCI. Here, we found an accuracy of 39.6% (sensitivity_MCI_ = 13.7%, sensitivity_AD_ = 41.0%, specificity = 60.7%).

*Results from extracting six microstates*

**
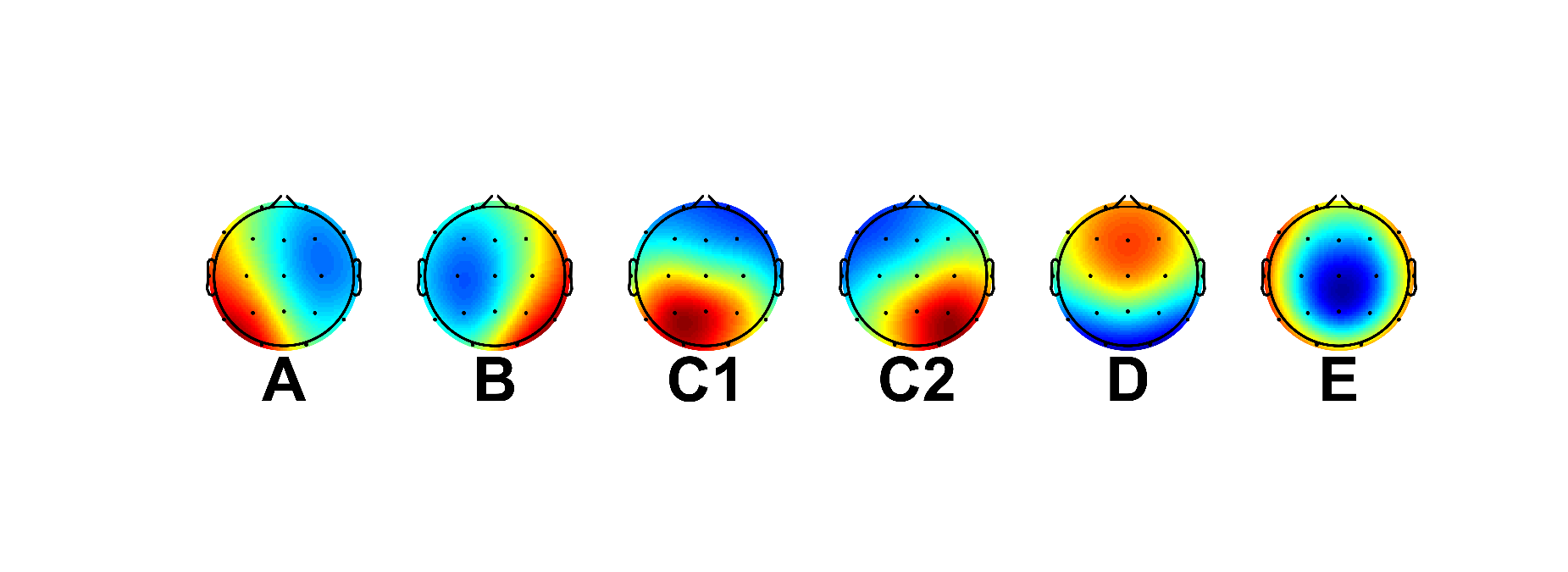
**

**Supplemental figure 5**: The global maps that were calculated based the aggregated dataset from all participants and were back-fitted to each of the EEG recordings. The first four microstates are labelled as A-D according to the literature in the field, and we labelled the third and fourth microstate as C1, and C2.

There was no significant difference in the total GEV between the three diagnostic groups (p-value = 0.886, F-value = 0.120) with an average GEV across groups of 51.57%.

*Prediction analysis*: We compared HC and the disease groups AD, and MCI. Here, we found an accuracy of 35.8% (sensitivity_MCI_ = 23.1%, sensitivity_AD_ = 34.2%, specificity = 48.1%).

*Conclusion*

In general, we find that there was no large difference in GEV between three and six microstates. Furthermore, we found that patients with AD had a global affection of microstates but there was a significant affection of microstate A when extracting both three, five, and six microstates. There results suggest that changes in microstate A is the hallmark of EEG microstate changes in AD. Lastly, we also performed the same prediction analysis as described under *Methods* and found that the accuracy was close to the results from four microstates but decreased when increasing the number of microstates.

**Results after removing the youngest 18 participants in the HC group**

After removing the 18 youngest HC, we still found a significant lower age as compared with AD and MCI (p-value <0.001).


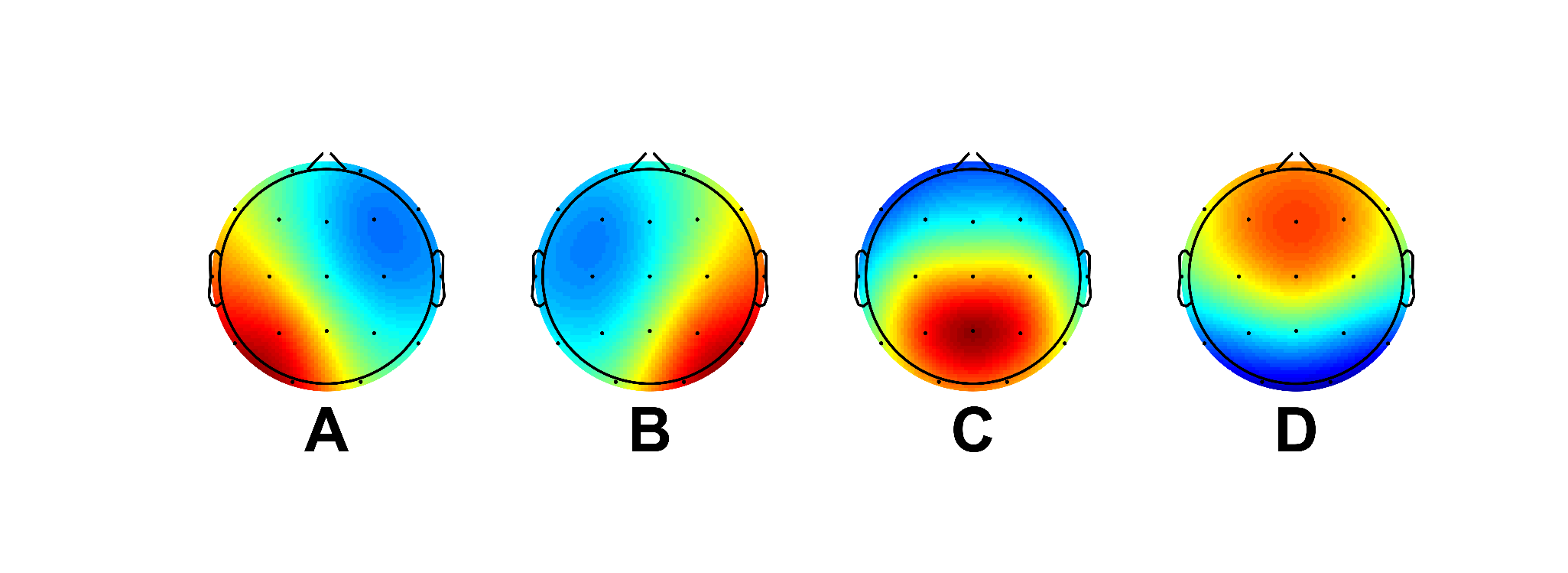


**Supplementary figure 6**: The global maps that were calculated based the aggregated dataset from all participants after removing the 18 youngest HC and were back-fitted to each of the EEG recordings.

For the analysis of the groups when removing the 18 youngest HC, we found an accuracy of 36.5% (sensitivity_MCI_ = 22.2%, sensitivity_AD_ = 36.8%, specificity = 50.4%). When investigating two classes, we found an accuracy of 62.8% (sensitivity = 66.7%, specificity = 59.0%) between AD and HC and an accuracy of 56.0% (sensitivity = 53.8%, specificity = 58.1%) between MCI and HC.

**Conclusion**

After removing the youngest 18 HC and thereby ending up with equal number of persons in each group, we did not find any large differences between the results above and the main analysis where the 18 youngest HC were not removed.
